# Impact of Research Educational Intervention on Knowledge, Attitudes, Perceptions, and Pharmacy Practices Towards Evidence-based Medicine Among Junior Pharmacists

**DOI:** 10.7759/cureus.2820

**Published:** 2018-06-18

**Authors:** Reem A Bahmaid, Mohammad Karim, Najwa Al-Ghamdi, Mohamad Al-Tannir

**Affiliations:** 1 Pharmacy, King Fahad Medical City, Riyadh, SAU; 2 Research Center, King Fahad Medical City, Riyadh, SAU

**Keywords:** ebm, educational intervention, knowledge, pharmacists

## Abstract

Background

Establishing evidence-based medicine (EBM) is important for pharmaceutical care services to be effective and for adding value to patient care. Increasing examples are illustrating that health professionals hold positive attitudes toward EBM. Nevertheless, their knowledge and skills are relatively insufficient. The objective of this study was to assess the impact of research educational intervention on knowledge, attitudes, perceptions, and pharmacy practices towards evidence-based medicine among junior pharmacists.

Methods

A one group pre-test/post-test quasi-experimental design was conducted on postgraduate junior pharmacy staff working or training at one of the three randomly selected tertiary care settings in Riyadh, Saudi Arabia. This study consisted of two phases. During the first phase, a structured questionnaire assessing the knowledge, perceptions, and attitudes of the participants regarding EBM, as well as basic biostatistics, epidemiology and the utilization of EBM, was administered. The second phase was scheduled to begin four weeks after the distribution of the educational materials, whereby the same questionnaire was redistributed among the same participants.

Results

Sixty-seven pharmacists participated in this study. The overall percentage mean score of correct responses of the study participants' knowledge was 37.0% in the pre-test compared to 44.4% in the post-test. The percentage mean score of correct responses for biostatistics and epidemiology and study design sections significantly increased after the study intervention (p < 0.001), (p = 0.02), respectively. Regarding the study participants' attitudes towards EBM, only one item, "Willingness to support the promotion of EBM implementation,” was statistically significantly higher in the post-test (61, 93.8%) participants compared to participants (53, 80.3%) in the pre-test, while "Possessing sufficient skills to implement EBM principles" was the only statistically significant item for the study participants' perceptions towards EBM in the pre-test compared to the post-test, (82.1%, 92.4%), respectively. Moreover, our results showed that 74.6% of the respondents were practicing EBM before the study intervention versus 81.5% after the intervention.

Conclusion

The results of this study reveal that comprehensive educational intervention might improve the knowledge, attitudes, and perceptions of EBM among pharmacists and encourage them to incorporate this into their everyday clinical practice.

## Introduction

Evidence-based medicine (EBM) is “the process of systematically finding, appraising, and using contemporaneous research findings as the basis for clinical decisions” [1]. The concept of EBM, more broadly, marks a shift among healthcare professionals from a traditional emphasis on actions based on the opinions of authorities to guide clinical practice to an emphasis on data-based, clinically relevant studies and research.

The practice of EBM involves four primary steps: formulating a clear question based on a patient problem, identifying relevant studies from the literature, critically appraising the validity and usefulness of the identified studies, and applying the findings in clinical practice [2]. Despite enthusiasm in the educational and research communities for EBM, the attitudes of practicing general internists about EBM have not been systematically investigated. Although EBM encourages the use of primary research studies, evidence-based clinical practice guidelines, and systematic overviews to inform treatment decisions, recent surveys have suggested that most physicians still rely heavily on the opinion of colleagues or consultants when making decisions [3-5].

The pharmacy profession has improved to include the provision of cognitive services, in addition to the traditional role of medication dispensing. Establishing EBM is important for pharmaceutical care services to be effective and for adding value to patient care. Pharmacists must accept and actively participate in the research needed to establish the required evidence-based pharmaceutical care. In a survey of pharmacists, the majority held a positive attitude towards evidence-based practices, which reflects the awareness of the pharmacists towards their profession in EBM [2].

Acknowledgment of EBM and the study of design, including understanding methods of evaluating, interpreting, and criticizing primary literature, is indeed not new among junior pharmacists but it is still gaining traction. Many studies have evaluated the perceptions of EBM among health care professionals [6-7]. Others have ascertained the knowledge on adverse effects associated with complementary medicines, as well as evaluating awareness of pharmacovigilance among pharmacists and other healthcare providers.

There are examples illustrating that health professionals hold positive attitudes toward EBM [8-11]. Nevertheless, their knowledge and skills about the implementation of EBM are relatively insufficient [11-14]. Moreover, measuring perception, knowledge, and the ability to evaluate, interpret, and criticize literature among junior pharmacists is still lacking [15]. The objective of this study was to assess the impact of educational intervention on the research knowledge, attitudes, perceptions, and practices towards EBM among junior and intern pharmacists.

## Materials and methods

Study design

A one group pretest-posttest quasi-experimental design was conducted from January 2017 to December 2017 at three tertiary care settings: King Fahad Medical City, King Faisal Specialist Hospital and Research Center and Prince Sultan Cardiac Center, Riyadh-Saudi Arabia.  

Study participants

All postgraduate junior pharmacy staff who were working or training at one of the three randomly selected tertiary care settings were eligible to participate in this study. The junior pharmacist represents those pharmacists who have been in practice for no more than one year or are still undergoing on-the-job training during the defined study period.

Recruitment

The study participants were randomly approached and invited to take part in this study by a trained research coordinator over a six-month period to reach the needed sample size.

Intervention and data collection procedure

This study consisted of two phases. During the first phase, a structured questionnaire assessing the knowledge, perceptions, and attitudes of the participants regarding EBM as well as basic biostatistics, epidemiology and the utilization of EBM was administered (pretest). Upon completion, the study results were interpreted. After that, the participants were provided with educational material. The content of the educational material was based on a review of the literature, covering the core element of EBM which is training in research related skills and study design [16,17]. The curriculum covered (i) basic biostatistics and (ii) basic epidemiology and study design. To facilitating learning, the participants were provided with hard copies of educational materials and asked to read the materials daily. Moreover, participants had received reminder twice a week to read the educational materials.

The second phase was scheduled to begin four weeks after the distribution of the educational materials, whereby the same questionnaire was re-distributed among the same participants (posttest). The questionnaire was developed based on the content of the educational materials that were provided to the participants and according to Downing’s recommendations for effective test development [18]. The questionnaire encompassed 4 sections. The first section is the knowledge section which included 19 multiple-choice question about basic biostatistics and basic epidemiology and study designs. The biostatistics part incorporated questions related to hypothesis testing and other questions related to descriptive and inferential statistics. Whereas, epidemiology and study designs part included questions to recognizing the appropriate study design and other questions related to basic epidemiology measurements, randomization, bias and confounding factors. To calculate the participants’ knowledge score, correct answers were given a score of 1 whereas, incorrect answers or unanswered question were given a score of 0. The total score of the correct answers reflected the participants’ knowledge level. The second section included 4 questions to identify the study participants' attitudes towards EBM. The attitudes questions were assessing their interest in learning or improving the skills necessary to incorporate EBM into practice, willingness to support the promotion of EBM implementation, decisions about patient care and need to increase the use of evidence in daily practice. The third section included 9 questions to identify the participants' perceptions towards EBM. A 5-point Likert scale was used for the attitudes' and perceptions' questions (strongly agree, agree, uncertain, disagree, and strongly disagree). The last section was asking participants about practicing EBM. Moreover, demographic characteristics encompassed gender, age and educational background (Pharm-D holders have mandatory clinical training for nine months before graduation while pharmacy degree holders only undergo four months of clinical training). Total years of clinical experience were also collected from the study participants.

The questionnaire was piloted and evaluated for its face validity by a panel of experts including physicians, researchers, and senior pharmacists. Some modifications in the questions were made after discussions with the panel of experts. Moreover, a pilot study was performed on 10 subjects to enhance the clarity of the questionnaire and was revised as per their comments.

Ethical consideration

The study was approved by the institutional review board of King Fahad Medical City. Participants who met the inclusion criteria and agreed to participate were asked to sign a consent form which informs participants about the purpose of the study and ensures that their personal information will be kept confidential.

Sample size calculation

The sample size was calculated by the Raosoft® sample size software calculator (Raosoft, Inc., Seattle, WA) with presumed 50% having knowledge of EBM among the study participants, a 95% confidence interval, and a 5% margin of error. This allowed us to calculate the required sample size of 67 participants.

Statistical analysis

All the contemporaneous research findings for EBM were based on clinical decisions. Thereby, the studied variables were of ordinal or nominal order which was presented in frequencies and percentages. Cronbach’s alpha test was applied to assess the internal consistency of EBM, scores, and another scale of measurement. The assessment questionnaire had Cronbach’s alpha = 0.96. Ordinal scale data was represented as a binary outcome, and a paired t-test analyzed the respective pooled Relationship Assessment Scores (RAS). All the inferences were drawn at 95% confidence interval (CI). The responses of the Likert scale were joined into combinations of (I) “strongly agree” and “agree” and (II) “uncertain”, “disagree”, and “strongly disagree”. Microsoft Excel® (Microsoft Corp., Redmond, WA) and the Statistical Package for the Social Sciences (SPSS) (IBM SPSS Statistics, Armonk, NY), version 22 software, were used for data analysis.

## Results

Sociodemographic characteristics of the study participants are presented in Table 1. The majority of participants were female (85.1%) and had less than one year of clinical experience (71.6%). Moreover, each the study participants had no previous formal training in EBM.

**Table 1:** Sociodemographic Characteristics of the Study Participants n: number; Pharm-D: pharmacy degree

Characteristics		n	(%)
Gender	Female	57	(85.1)
	Male	10	(14.9)
Age (years)	22-30	66	(98.5)
	31-40	1	(1.5)
Academic degree	Bachelor Pharmacy	21	(31.3)
	Pharm-D	46	(68.7)
Total years of clinical experience	< 1	48	(71.6)
	1-2	16	(23.9)
	> 2	3	(4.5)

Participants' knowledge of the basic biostatistics, epidemiology, and study design

After calculating the knowledge score for every single section before and after the study intervention, our results showed that the overall percentage mean score of correct responses by the study participants was 37.0% in the pre-test compared to 44.4% in the post-test.

The percentage mean score of correct responses for the biostatistics section was 33.9% in the pre-test versus 43.5% in the post-test (p < 0.001). In epidemiology and study design section, the percentage mean score of correct responses significantly increased after the study intervention (p = 0.02) (Table 2).

**Table 2:** Pre-test and Post-test of the Participants' Knowledge of the Basic Biostatistics, Epidemiology, and Study Design ∗p-value is statistically significant. SD: standard deviation

	Pre-test	Post-test	p-value
Mean basic biostatistics score of correct responses (% mean score) ± SD	2.4 (33.9%) ± 1.3	3.0 (43.5%) ± 1.3	< 0.001*
Mean epidemiology and study design score of correct responses (% mean score) ± SD	4.7 (38.8%) ± 1.4	5.4 (44.9%) ± 2.1	0.02*
Overall mean score of correct responses (% mean score) ± SD	7.0 (37.0%) ± 2.04	8.4 (44.4%) ± 2.9	< 0.001*

Participants' attitudes, perceptions, and practices towards EBM

Regarding the study participants' attitudes towards EBM, only one item, "Willingness to support the promotion of EBM implementation”, was statistically significantly higher in the post-test (61, 93.8%) participants compared to 53 (80.3%) participants in the pretest (p = 0.021). While "Possessing sufficient skills to implement EBM principles" was the only statistically significant item for the study participants' perceptions towards EBM in the pre-test compared to the post-test, (82.1%, 92.4%), respectively, (p = 0.031) (Table 3). Our results after asking participants about practicing EBM indicated that 74.6% of respondents were practicing EBM before the study intervention versus 81.5% after the intervention.

**Table 3:** Attitudes and Perceptions of the Study Participants Towards Evidence-based Medicine ∗p-value is statistically significant. EBM: evidence-based medicine

Items	Agree Pre-test n (%)	Agree Post-test n (%)	p-value
Attitudes			
Interested in learning or improving the skills necessary to incorporate EBM into my practice	62 (92.5)	59 (89.4)	0.754
Willingness to support the promotion of EBM implementation	53 (80.3)	61 (93.8)	0.021*
EBM helps me make decisions about patient care	62 (92.5)	59 (89.4)	0.754
Need to increase the use of evidence in my daily practice	58 (87.9)	57 (86.4)	1.000
Perceptions			
EBM improves the quality of patient care	62 (93.9)	61 (92.4)	1.000
Literature and research findings are useful in daily practice	54 (80.6)	53 (80.3)	1.000
Having sufficient knowledge to implement EBM principles	56 (83.6)	59 (89.4)	0.375
Possessing sufficient skills to implement EBM principles	55 (82.1)	61 (92.4)	0.031*
Application of EBM is necessary in the practice of pharmacy	57 (86.4)	55 (83.3)	0.549
The adoption of EBM places an unreasonable demand on a pharmacist	6 (9.7)	11 (16.9)	0.277
EBM does not take into account the limitations of my practice setting	17 (26.2)	16 (24.2)	0.804
My salary rate will increase if I incorporate EBM in my practice	16 (24.6)	23 (35.4)	0.118
Strong evidence is lacking to support most of the interventions I use in practice	19 (29.7)	18 (28.1)	0.815

All items in the questionnaire were studied to assess the association between the sociodemographic characteristics, research knowledge, attitudes, perceptions, and practices among the participants before and after the study intervention. Only the RAS pre-test EBM percentage mean score was significantly higher among Pharm-D (pharmacy degree) holders 85.5 ± 19.1 compared to 73 ± 29.1 among bachelor pharmacy degree holders (Table 4).

**Table 4:** Sociodemographic Characteristics and Participants' Research Knowledge Data are presented as percentage mean score and ± standard deviation. ∗p-value is statistically significant. n: number; Pharm-D: pharmacy degree; RAS: Relative assessment scale

Variable			p-value
Gender	Female (n = 57)	Male (n = 10)	
RAS Pre-test	81 ± 24.3	85 ± 16.6	0.617
RAS Post-test	91.5 ± 13.2	90 ± 14	0.743
Academic level	Pharmacy	Pharm-D	
RAS Pre-test	73 ± 29.1	85.5 ± 19.1	0.040*
RAS Post-test	90.8 ± 13.8	91.5 ± 13.1	0.859
Total years of clinical experience	< 1 yr.	≥ 1 yrs.	
RAS Pre-test	81.2 ± 24.5	82.5 ± 20.4	0.850
RAS Post-test	90.9 ± 13.9	92.1 ± 11.6	0.750

## Discussion

Educational intervention is a common way to spread EBM [19-22]. In our study, although the overall research knowledge score of the study participants has improved significantly after the study intervention, the percentage mean score of correct responses was low. This might be explained by the fact that research training is not required for pharmacists in Saudi Arabia and most staff continue to practice based on what they learned in school and their practice experiences.

Our results provide important evidence in the plan for spreading the knowledge and implementation of EBM as it encourages self-based learning. Previous studies reported that cooperative tasks enhance our ability to learn more than individual teaching [23-24]. Our results revealed that establishing comprehensive educational intervention can enhance knowledge and EBM practices among the study participants.

Previous studies reported that health professionals have positive attitudes towards EBM [8, 12]. A study conducted on pharmacists showed that 90% have positive attitudes towards EBM and 84% thought research findings were an important daily practice; these results reflect the awareness of the pharmacists towards their profession in EBM [2]. Likewise, regarding the participants' attitudes and perceptions, our results showed that the study participants were more willing to support the promotion of EBM implementation and perceived that they possessed sufficient skills to implement EBM principles after the intervention. Leaders can power this attitude by providing chances for practices, which in turn might positively enhance the knowledge towards EBM.

Moreover, our results showed that the research knowledge among participants holding Pharm-D degrees was significantly higher compared to bachelor pharmacy degree holders. This might be explained by the fact that Pharm-D holders have mandatory clinical training for nine months before graduation, while pharmacy degree holders have clinical training for only four months.

The results of this study provide leaders and educators guidance to promote knowledgeable attitudes, perceptions, and practices related to EBM. Attaining baseline information about EBM among the staff permits an organization to develop educational activities and process modifications to fruitfully include EBM into daily practice, as the staff’s knowledge, attitudes, perceptions, and practices affect the achievement of any initiatives to implement EBM.

The strengths of this study are that the study was conducted in tertiary care settings and the study participants in pre-testing and post-testing were the same subjects. However, the study had some limitations as it is a self-report survey, not an audit of actual practice and did not include a control group. Furthermore, our study explored the short-term effects of the intervention on the study participants' research knowledge, attitudes, and practices of EBM. Despite the limitation, this study aims to stimulate more research on this critical issue, especially long-term follow-up and controlled studies of EBM educational intervention on the study participants' knowledge, attitudes, and practices.

## Conclusions

In conclusion, the results of this study reveal that comprehensive research educational intervention might improve the knowledge, attitudes, and perceptions of EBM among pharmacists and encourage them to incorporate this into their everyday clinical practice.

## Appendices

Questionnaire

General Information

Serial # ___________                                    Date: ________________

Note: To be filled by junior pharmacy staff and intern pharmacists:

Gender:           Male           Female

Nationality:         Saudi         Non – Saudi

Age:        20 – 30         31 – 40

Academic Level:         Bachelor’s         Pharm-D          Resident         Intern (Bachelor’s)         Intern (Pharm-D)

Years since pharmacy school graduation

        < 1                1 – 2            > 2

This section of the questionnaire inquires about your evidence-based medicine (EBM) knowledge and perceived benefits and limitations of EBM.

Please answer the following questions to the best of your capability about EBM.

1)   Have you heard of EBM?

None           a little          a fair amount           a lot

2)   Do you believe EBM is important for improving patient care quality?

Strongly Yes            Neutral             Strongly No

3)   Are you willing to support the promotion of EBM implementation?

Yes             No          I Do Not Know

4)   Do you have sufficient knowledge to implement EBM principles?

 None             a little             a fair amount           a lot

5)   Do you possess sufficient skills to implement EBM principles?

 None             a little            a fair amount           a lot

6)   In the past two years, have you searched for relevant evidence in the literature to resolve your clinical questions, and then applied the findings to clinical decision-making after critical appraisal?

Yes             No            To some degree

This section of the questionnaire inquires about attitudes toward and use of EBM.

1)   Application of EBM is necessary for the practice of pharmacy.

Strongly Disagree            Disagree             Neutral            Agree            Strongly Agree

2)   Literature and research findings are useful in my day-to-day practice.

Strongly Disagree            Disagree             Neutral            Agree            Strongly Agree

3)   I need to increase the use of evidence in my daily practice.

Strongly Disagree           Disagree               Neutral           Agree             Strongly Agree

4)   The adoption of EBM places an unreasonable demand on a pharmacist.

Strongly Disagree           Disagree              Neutral          Agree              Strongly Agree

5)   I am interested in learning or improving the skills necessary to incorporate EBM into my practice.

Strongly Disagree           Disagree              Neutral          Agree               Strongly Agree

6)   EBM improves the quality of patient care.

Strongly Disagree          Disagree              Neutral           Agree               Strongly Agree

7)   EBM does not take into account the limitations of my practice setting.

Strongly Disagree          Disagree               Neutral           Agree              Strongly Agree

8)   My salary rate will increase if I incorporate EBM into my practice.

Strongly  Disagree         Disagree               Neutral           Agree              Strongly Agree

9)   Strong evidence is lacking to support most of the interventions I use in practice.

Strongly Disagree           Disagree               Neutral         Agree              Strongly Agree

10)   EBM helps me make decisions about patient care.

Strongly Disagree         Disagree               Neutral           Agree              Strongly Agree

Basic Biostatistics, Epidemiology, and Study Design Test Instrument

Please choose the best answer from each of the following questions:

A study wishes to assess birth weight characteristics in a population. Which of the following variables describe the appropriate measurement scale or type?

A.   Discrete

B.   Continuous

C.   Ordinal

D.   Nominal

E.   Dichotomous

A normal distribution curve is determined by which of the following:

A.   Mean and Sample Size

B.   Mean and Standard Deviation

C.   Range and Sample Size

D.   Range and Standard Deviation

E.   Mean and Range

An analysis of patients according to their spoken language reveals that 40% speak Arabic, 55% English, and 0.5% French. These figures would best be represented graphically with which of the following:

A.   Venn Diagram

B.   Cumulative Frequency

C.   Normal Curve

D.   Histogram

E.   Pie Chart

The following data represent the length of hospitalization (in weeks) for five patients:  3, 4, 5, 6, and 20 days. Which of the following is the best measure of central tendency for this set of data?

A.   Mean

B.   Mode

C.   Range

D.   Median

E.   Standard Deviation

In preparation for a national examination, 200 medical students complete 100 questions in a practice test. Each student answered between 35 and 59 questions correctly. The number of correct answers per student was distributed normally. What is the range of questions answered correctly?

12

24

36

65

94

The mean of four numbers is 71.5 if three of the numbers are 58, 76, and 88. Which of the following would be the fourth value?

64

60

76

82

28

A study that examined the relationship between birth weight and salary at age 50 found the r-value to be 0.8. This value can be interpreted as which one of the following?

Birth weight caused a high salary at age 50

Low birth weight caused a high salary at age 50

There is a statistically significant relationship between these two variables

There is no association between these two variables

None of the above

A cross-sectional study is a suitable study design to measure which of the following

A.   Prevalence rate

B.   Odds ratio

C.   Relative Risk

D.   Incidence Rate

E.   Cumulative Incidence

In a cohort study designed to determine an association between measles, mumps, rubella vaccination, and autism, the investigator reports the relative risk of autism in the vaccinated group compared to the unvaccinated group as 0.92 (95%  CI = 0.65 – 1.07). Which of the following p-values is consistent with these reported findings?

A p-value of less than 0.05

A p-value of less than 0.01

A p-value of greater than 0.05

A p-value of greater than 0.01

Any systematic error in the design, conduct, or analysis of a study that results in a mistaken estimate of an exposure effect on the risk of disease is called:

Confounding

Bias

Interaction

Stratification

To determine if smoking is associated with lung cancer, data from 40 patients with lung cancer were collected. These patients were matched for age, sex, and race to 40 patients without lung cancer. The hospital charts of these patients were then reviewed. This study design typically is known as:

A.   Cross-sectional Study

B.   Concurrent Cohort Study

C.   Case-Control Study

D.   Retrospective Cohort Study

E.   Randomized Controlled Trial

A 39-year-old man presents with a mild sore throat, fever, malaise, and headache is treated with penicillin for the presumed streptococcal infection. He returned after a week with hypertension, fever, rash, and abdominal pain. He responded favorably to Chloramphenicol after a diagnosis of Rocky Mountain spotted fever is made. Select the study design that is most appropriately illustrated above.

Case Series

Case-Control Study

Clinical Trial

Cohort Study

Case Report

The inability to link exposure to disease in particular individuals and the inability to control for confounding variables are two limitations of which study design?

Case-Control Studies

Correlation Studies

Cohort Studies

Both (A) and (B)

Both (B) and (C)

The major source of bias in clinical trials is/are:

A.   Attrition (loss) during follow-up

B.   Non-compliance with assigned procedures among participants

C.   Lack of randomization of the subject to exposure groups

D.   Both (A) and (C)

A clinical trial in which neither the subjects nor the investigators know whether the actual treatment or a placebo is being administered is an example of which of the following:

Double-blinded clinical trial

Randomized clinical trial

Randomized clinical trial with a double placebo

Double treatment clinical trial

Controlling for selection bias

A case-control study is performed to judge whether a drug is associated with an increased incidence of early miscarriage. The final analysis showed that the odds ratios (OR) for miscarriage with drug exposure is 1.3 (95% confidence interval (CI) = 0.9 – 1.7). Which one of the following provides a correct description of the result? 

The drug increases the risk of miscarriage by 70%.

The drug increases the risk of miscarriage by 30%.

The drug decreases the risk of miscarriage by 10%.

The drug is not associated with an increased risk of miscarriage

Cohort studies are thought to provide better information than case-control studies with regard to the causal association between an exposure and a disease because:

Non-differential misclassification bias does not affect cohort studies

One can more clearly establish that exposure precedes disease

Cohort studies are better at assessing rare exposures

Larger sample sizes required for cohort studies provide better power to detect the true association between exposure and disease

Less affected by recall bias

Researchers want to assess if there is an association between cigarette smoking and stroke. Which of the following best study design should be sued to assess for this association?

Case Series

Cross-sectional Study

Prospective Cohort

Randomized Controlled Trial

Interventional Study
